# Selective autophagy as a therapeutic target for neurological diseases

**DOI:** 10.1007/s00018-020-03667-9

**Published:** 2020-10-16

**Authors:** Weilin Xu, Umut Ocak, Liansheng Gao, Sheng Tu, Cameron J. Lenahan, Jianmin Zhang, Anwen Shao

**Affiliations:** 1 Department of Neurosurgery, Second Affiliated Hospital, School of Medicine, Zhejiang University, Hangzhou, China; 2 Department of Emergency Medicine, Bursa Yuksek Ihtisas Training and Research Hospital, University of Health Sciences, 16310 Bursa, Turkey; 3 Department of Emergency Medicine, Bursa City Hospital, 16110 Bursa, Turkey; 4 State Key Laboratory for Diagnosis and Treatment of Infectious Diseases, Collaborative Innovation Center for Diagnosis and Treatment of Infectious Diseases, First Affiliated Hospital, College of Medicine, Zhejiang University, Hangzhou, 310009 Zhejiang China; 5 Brain Research Institute, Zhejiang University, Hangzhou, China; 6 Collaborative Innovation Center for Brain Science, Zhejiang University, Hangzhou, China; 7 Burrell College of Osteopathic Medicine, Las Cruces, NM USA

**Keywords:** Stroke, Alzheimer’s disease, Parkinson’s disease, Neuroprotection, Macroautophagy, Autophagy receptor

## Abstract

The neurological diseases primarily include acute injuries, chronic neurodegeneration, and others (e.g., infectious diseases of the central nervous system). Autophagy is a housekeeping process responsible for the bulk degradation of misfolded protein aggregates and damaged organelles through the lysosomal machinery. Recent studies have suggested that autophagy, particularly selective autophagy, such as mitophagy, pexophagy, ER-phagy, ribophagy, lipophagy, etc., is closely implicated in neurological diseases. These forms of selective autophagy are controlled by a group of important proteins, including PTEN-induced kinase 1 (PINK1), Parkin, p62, optineurin (OPTN), neighbor of BRCA1 gene 1 (NBR1), and nuclear fragile X mental retardation-interacting protein 1 (NUFIP1). This review highlights the characteristics and underlying mechanisms of different types of selective autophagy, and their implications in various forms of neurological diseases.

## Introduction

### Neurological diseases

The nervous system is regarded as our body’s command center. any impairment or interruption in the nervous system would induce a dysfunctional physiological state, named neurological diseases [1]. Neurological diseases can be categorized as acute injuries (e.g., ischemic or hemorrhagic stroke, spinal cord injury, and traumatic brain injury), chronic neurodegeneration [e.g., Alzheimer’s disease (AD) and Parkinson’s disease (PD)], and others (e.g., brain tumors, center nervous system infectious disease, etc.) [2, 3]. The underlying molecular mechanisms involve neuronal apoptosis, neuroinflammation, oxidative stress, autophagy, etc. [4, 5]. For example, deposits of massive amyloid-β peptide lead to neuroinflammation and oxidative stress in patients with AD, which finally cause neuronal apoptosis [6–8]. Furthermore, growing evidence suggests that mitochondrial dysfunction, redox imbalance, massive deposits of aberrant proteins (i.e., α-synuclein), and damage of the ubiquitin–proteasome system contribute to the pathophysiology of PD [9, 10]. Moreover, stroke, defined as a lack of blood supply to the brain or the presence of blood spreading into the brain and subarachnoid space, would cause dysfunction of the mitochondria, endoplasmic reticulum, peroxisome, etc., further introducing oxidative stress and inflammation, and finally causing cell death [11, 12]. Therefore, strategies and programs to treat neurological diseases could significantly reduce the global burden.

### Autophagy

Autophagy was first described by Sam L. Clark Jr. as ‘self-eating’ in 1957, which was confirmed by Christian de Duve who found the debris of intracellular organelle structures within lysosomes [13, 14]. Autophagy can be activated by stress (stroke, trauma, etc.) or nutrient deprivation. The main physiological functions of autophagy not only include degradation or recycling of long-lived proteins, but also the elimination of dysfunctional or broken organelles, such as the mitochondria, peroxisomes, or ribosomes [15]. There are three different types of autophagy reported in mammalian cells according to their method of substrate delivery: macroautophagy, microautophagy, and chaperone-mediated autophagy [16]. On the other hand, autophagy could also be regarded as a non-selective pathway for recycling nutrition, but as a selective way to remove dysfunctional and damaged organelles [17].

#### Macroautophagy

**Fig. 1:**
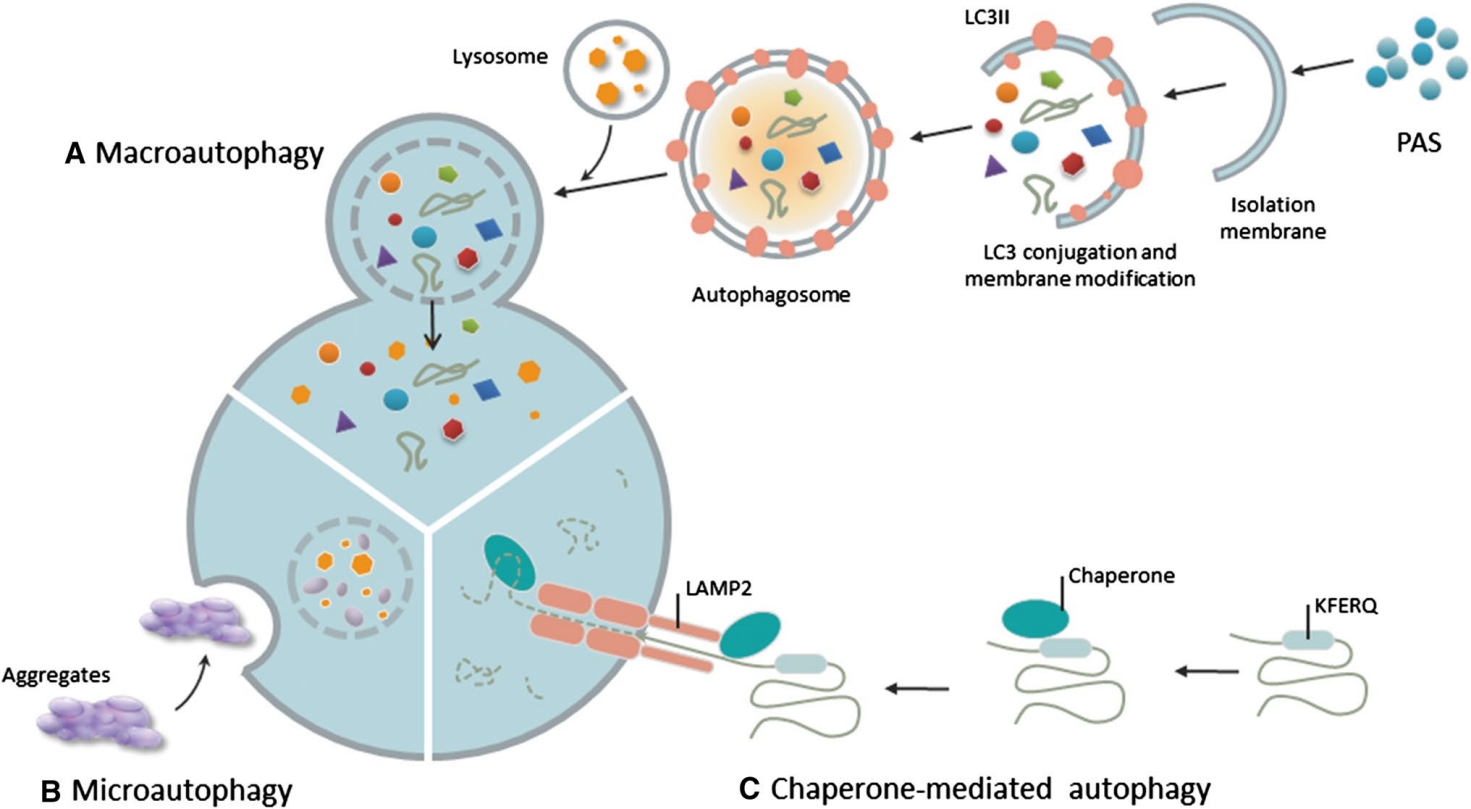
Three types of autophagy: macroautophagy, microautophagy, and chaperone-mediated autophagy

There remains much to be uncovered regarding autophagy-related genetic proteins, and their involvement in different steps of autophagy in yeast, most of which are conserved in mammals (Fig. 1) [18, 19]. The process of autophagy starts with the assembly of phagophore assembly site (PAS). Then, the UNC51-like kinase (ULK) complex assembles to the PAS [20]. After that, the class III phosphatidylinositol 3-kinase (PI3K III) complex helps to form the nucleation of autophagy [21]. Following nucleation, the formation of an Atg5–Atg12–Atg16-like 1 (Atg16L1) complex is required to facilitate cargo recognition and autophagosome membrane elongation [22].

#### Microautophagy

Microautophagy is a catabolic process, in which the dysfunctional or superfluous proteins and organelles are delivered directly to the endosomal/lysosomal lumen (Fig. 1) [23]. However, in mammalian cells, the detailed molecular mechanisms engaged in the process of microautophagy are still not well understood. However, the process of microautophagy largely depends on the endosomal sorting complexes required for transport (ESCRT) I and III systems and the protein chaperone, hsc70 [24].

#### Chaperone-mediated autophagy (CMA)

The main function of CMA is to degrade the proteins with a KFERQ motif [25]. Nearly 30% of cytosolic proteins contain the KFERQ motif [26]. The lysosome-targeted proteins with a KFERQ motif are first transferred to a lysosome-associated membrane protein 2 (LAMP2A)-containing complex on the lysosomal membrane with the help of chaperones (heat shock cognate 70 (hsc70), heat shock protein 70 (Hsp70)), and co-chaperones, including HSP40, Hsc 70-interacting protein (HIP), Hsp70–Hsp90 Organizing Protein (HOP), Hsp90. Then, the target proteins are unfolded and degraded under the assistance of a complex of proteins in the lysosomal lumen, including Hsc70 [27].

## Selective autophagy

**Fig. 2:**
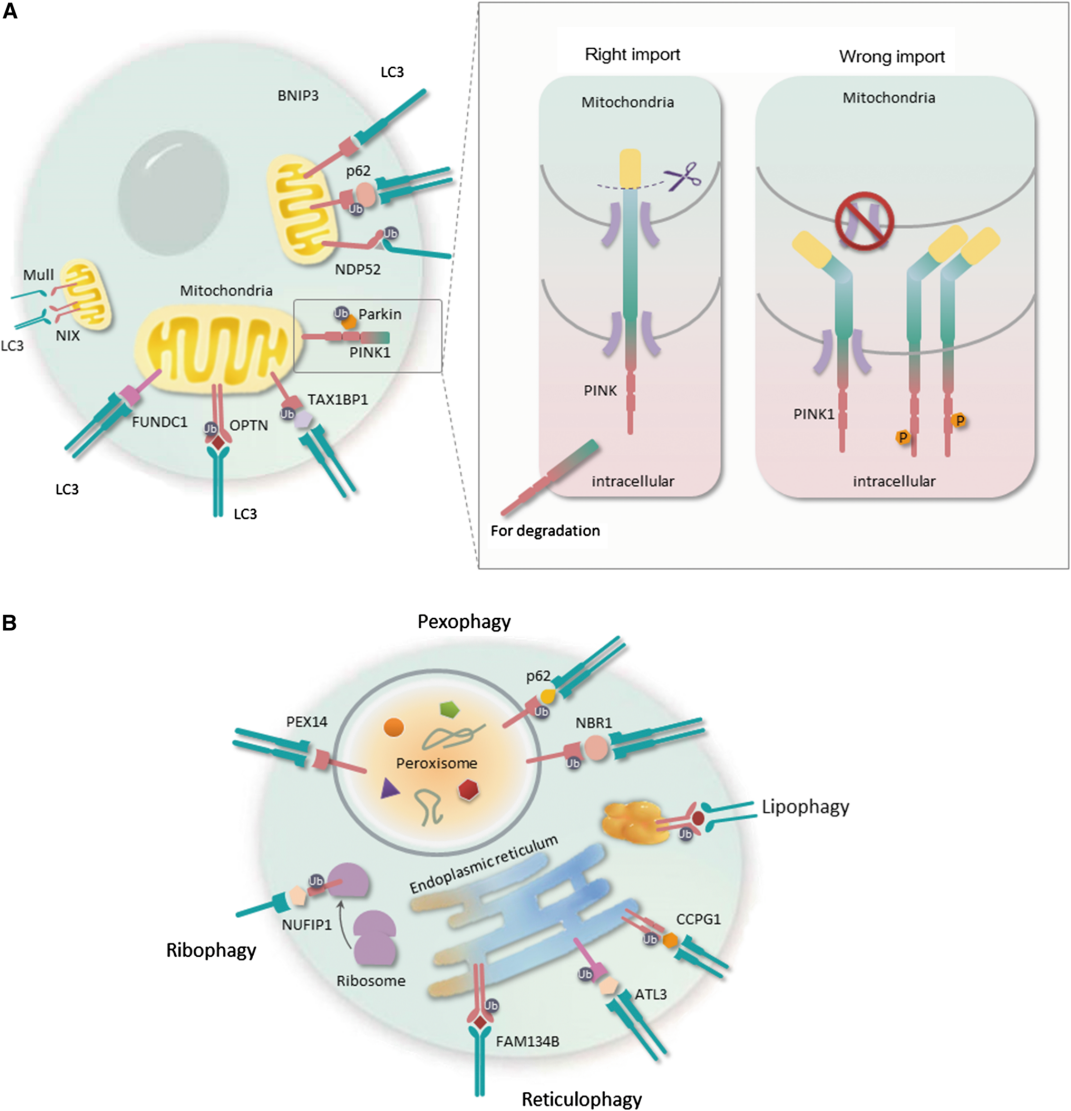
The autophagy process of five common types of selective autophagy: **a** the process and regulatory mechanism of mitophagy; **b** the regulatory mechanism of pexophagy, ER-phagy, ribophagy, and lipophagy

Selective autophagy (mitophagy, pexophagy, ER-phagy, ribophagy, and lipophagy) is a process in which a lysosomal-targeted cargo is selectively recognized and degraded, relying on receptor proteins that bind Hsp70–Hsp90 Organizing Protein (HOP) [28] (Fig. 2). Many proteins contribute to the process of selective autophagy. For example, when mitophagy is triggered, Parkin is phosphorylated and activated by PTEN-induced kinase 1 (PINK1), which can further activate and build ubiquitin chains, while autophagy receptors, such as nuclear domain 10 protein 52 (NDP52), SQSTM1 (p62), optineurin (OPTN), and Tax1-Binding Protein 1 (TAX1BP1), target dysfunctional mitochondria to autophagosomal membranes [29, 30]. In pexophagy, peroxin 5 (PEX5) and PEX14 are the peroxisome resident proteins that initiate the pexophagy process [31]. Additionally, selective autophagy is also involved in many pathophysiological processes. For example, the improvement of mitophagy or pexophagy could alleviate the inflammation and oxidative stress after various cellular stresses, and ultimately prevent the cells from dying [32, 33]. The development of neurological diseases leads to the production of many dysfunctional and superfluous organelles (mitochondria, endoplasmic reticulum, peroxisome, etc.), which would introduce severe conditions, such as oxidative stress, neuroinflammation, blood–brain barrier (BBB) disruption, and neuronal apoptosis [34, 35]. Therefore, selective clearance of these organelles is critical to improving neurological functions in patients with neurological diseases.

### Mitophagy

#### General introduction of mitochondria and mitophagy

Any disturbance or impairment of the mitochondria would lead to its dysfunction, which would then result in a sharp increase of reactive oxidative species (ROS). Redundant ROS would promote the release of pro-apoptotic factors and finally cause cell death [36, 37]. Mitophagy, the selective degradation of dysfunctional mitochondria defined by Lemasters in 2005, is essential for maintaining cell survival [38]. Furthermore, mitophagy is a process of macroautophagy, and involves three key steps: (1) assembly of phagophore assembly site (PAS); (2) formation of mitophagosome by targeting and engulfment of dysfunctional mitochondria; (3) formation of mitolysosome by fusing with lysosome [39–41]. Until now, most of the studies focused on exploring the molecular mechanisms to understand how phagophores are formed, and how dysfunctional mitochondria are recognized.

Many proteins are reportedly involved in the process of mitophagy. One of the most important proteins is PINK1, which is a mitochondrially localized kinase. The main function of PINK1 is to sense the damage of mitochondria, then activate Parkin, and help it translocate from the cytoplasm to the damaged mitochondria [42–45]. Parkin is an ubiquitin ligase, and normally remains in a “closed” state by hiding the enzyme domain [46]. When mitophagy is triggered, PINK1 will phosphorylate and activate Parkin, which can further activate and build ubiquitin chains. Another group of important proteins is autophagy receptors (Table 1), which assist the autophagy machinery in selectively targeting the mitochondria. These receptors share two important regions to direct mitochondria to autophagy machinery: LC3-interacting regions (LIR) and ubiquitin-binding domains (UBDs). As of now, five autophagy receptors have been reported, including NDP52, OPTN, p62, TAX1BP1, and NBR1 [47, 48]. However, of these five receptors, only NDP52 and OPTN are essential to initiate mitophagy, while others, such as p62, TAX1BP1, and NBR1, have a minor role in mitophagy [49]. NDP52 and OPTN not only target dysfunctional mitochondria to autophagosomal membranes, but also facilitate the formation of autophagosomal membranes by recruiting key factors, including ULK1, double FYVE domain-containing protein (DFCP1), WD Repeat Domain, Phosphoinositide Interacting 1 (WIPI1), etc. [49]. With the exception of the ubiquitination pathway, mitophagy can be initiated by mitophagy receptors, which target damaged mitochondria directly to autophagosomes for further degradation. Mitophagy receptors include NIP3-like protein X (NIX/Bnip3L), BCL2-like 13 (Bcl2L13), BCL2/adenovirus E1B interacting protein 3 (Bnip3), autophagy/Beclin 1 regulator 1 (AMBRA1), FUN14 domain-containing 1 (FUNDC1), and cardiolipid [39, 50, 51].

**Table 1:** The selective autophagy receptors

Selective autophagy	Receptors	Refs.
Mitophagy	Nuclear domain 10 protein 52 (NDP52)	[93]
	Optineurin (OPTN)	[93]
	Neighbor of BRCA1 gene 1 (NBR1)	[91, 92]
	SQSTM1(p62)	[86, 95]
	TAX1BP1	[93]
	BCL-2-like protein 13(BCL2L13)	[131]
	BCL2/adenovirus E1B 19 kDa interacting protein 3(BNIP3)	[134, 137, 333]
	FUN14 domain-containing 1 (FUNDC1)	[135]
	NIP3-like protein X (NIX)	[101]
Pexophagy	NBR1	[177]
	SQSTM1 or p62	[175, 191, 192]
Ribophagy	Nuclear fragile X mental retardation-interacting protein 1 (NUFIP1)	[200]
ER-phagy (reticulophagy)	Family with sequence similarity 134, member B (FAM134B)	[214, 215]
	Reticulon 3 (RTN3)	[208]
	Cell-cycle progression gene 1 (CCPG1)	[210]
	Atlastins 3 (ATL3)	[211, 213]

*NDP52* nuclear domain 10 protein 52; *OPTN* optineurin; *OPTN* optineurin; *NBR1* neighbor of BRCA1 gene 1; *NUFIP1* nuclear fragile X mental retardation-interacting protein 1; *CCPG-1* cell-cycle progression gene 1; *NIX* NIP3-like protein X; *FAM134B* family with sequence similarity 134, member B; *RTN3* reticulon 3; *ATL3* Atlastins 3; *FUNDC1* FUN14 domain-containing 1; *Bcl2L13* BCL2-like 13

Mitophagy could be triggered by various stimuli, such as starvation, hypoxia, stroke, or development. Considering the different physiological context of mitophagy, it can be categorized into three different types: basal, programmed, and stress-induced. Basal mitophagy means that the cells would degrade old or abnormal mitochondria under normal physiological conditions [52, 53]. Mitophagy that occurs in different cell types during development is considered ‘Programmed mitophagy’ [54–56]. Stress-induced mitophagy refers to the acute degradation of mitochondria as a result of severe extracellular stress [57].

#### Molecular pathways of mitophagy

Mitophagy pathways are classified as PINK1–Parkin-mediated and Parkin-independent (Fig. 2a).

##### PINK1–Parkin-mediated mitophagy

PINK1–Parkin-mediated mitophagy depends on the ubiquitination pathway [58], and is initiated with the activation of PINK1. PINK1 functions to sense mitochondrial damage signaling. Normally, PINK1 is delivered into the mitochondria with the help of TOM and TIM complexes, which are the inner and outer membrane translocases [59]. The N-terminal of PINK1, which is located on the inner membrane, would be cleaved by proteases [60–63], and the C-terminal is released back to the cytosol, and degraded in an N-end manner [64]. Therefore, the successful import of PINK1 maintains PINK1 at reduced activity. However, membrane potential dissipation prevents the importation of PINK1 into the mitochondria, disrupting the stability of PINK1 [57, 65, 66]. Next, PINK1 is activated via autophosphorylation [67–69], dimerization [70], and accumulation [59].

In healthy mitochondria, Parkin closes its enzyme domain via intramolecular interaction. To fully activate Parkin, functional PINK1 must complete two important phosphorylation processes, one is S65 in the Ubl domain of Parkin [71, 72] and another is an analogous S65 residue on ubiquitin (referred herein as pUb) [73–75]. The phosphorylation by PINK1 changes Parkin’s conformation, facilitates its interaction with mitochondria, and activates its E3 ligase activity [76, 77]. Afterwards, Parkin acts as an ubiquitin enzyme that works on the proteins of the mitochondrial outer membrane. PINK1 phosphorylates Poly-Ub chains, which act as an ‘eat me’ signal for further recognition.

Phosphorylated poly-Ub is recognized by autophagy receptors (p62, OPTN, etc.), which can promote the formation of the autophagosome by binding with LC3. TBK1 reportedly facilitates OPTN binding to Ub chains by phosphorylating OPTN and promotes the efficacy of mitochondrial clearance [78]. Moreover, OPTN and NDP52 can promote the synthesis of autophagosomal membranes through the recruitment of some key components of autophagosome biogenesis (WIPI1, ULK1, and DFCP1) [49]. In a recent study, Abudu et al. showed that NIPSNAP1 (nipsnap homolog 1) and NIPSNAP2, which are considered mitochondrial matrix proteins, act as “eat me” signals for damaged mitochondria to maintain sustained recruitment of SQSTM1-like receptors (SLRs) to ensure efficient mitophagy [79].

##### Parkin-independent mitophagy

Surmounting evidence showed that other ubiquitin E3 ligases, such as ariadne RBR E3 Ubiquitin Protein Ligase 1 (ARIH1), Gp78, siah E3 Ubiquitin Protein Ligase 1 (SIAH1), and mitochondrial E3 Ubiquitin Protein Ligase 1 (MUL1), participate in promoting mitophagy, with the exception of Parkin [80–84]. These ubiquitin E3 ligases perform their functions by interacting with the mitochondrial membrane, generating ubiquitin chains, and promoting the recruitment of autophagy receptors (OPTN, NDP52, p62, etc.). These receptors then interact directly with LC3 and attach Ub-tagged organelles into autophagosomes [49].

##### Role of autophagy receptors in mitophagy

Moreover, some ubiquitin-independent mitochondrial proteins, such as BCL2L13, NIX, BNIP3, and FUNDC1, interact directly with LC3 and GABARAP on autophagosomal membrane without ubiquitination, and mediate mitophagy [85]. BCL2L13 is a functional homologue of Atg32 in mammals with an LIR motif, which interacts directly with LC3 to promote mitophagy in a Parkin-independent manner [86]. Besides, other proteins, such as NIX, BNIP3, and FUNDC1, are outer mitochondrial membrane proteins, and act as mitophagy receptors, which mediate mitochondrial clearance in response to different mitochondrial stresses. The NIX plays an especially important role in programmed mitophagy during differentiation [54–56, 87]. NIX-deficient cells accumulate mitochondria, leading to increased apoptosis and developmental defects [88]. LIR motif phosphorylation enhances NIX association with LC3 under stress conditions [89]. Although the signaling cascade of NIX-mediated mitophagy is not yet determined, Rheb, a small GTPase, may be involved, as its mitochondrial localization and physical interaction with NIX regulate mitochondrial removal and maintenance of energy metabolism [90].

Different from other receptors, BNIP3 participates in the regulation of mitochondrial dynamics by inducing mitochondrial fission through optic atrophy 1 (OPA1) disassembly and release, and by recruiting dynamin-related protein (DRP1) to mitochondrial outer membrane [91, 92]. BNIP3 mediates PINK1 stabilization by inhibiting its proteolytic cleavage [93]. Both NIX and BNIP3 sustain mitochondrial homeostasis through regulation of Parkin recruitment, suggesting crosstalk between mitophagy receptors and the PINK1–Parkin pathway [94].

FUNDC1 acts as a conserved mitophagy receptor and mediates mitophagy when there is a deficiency in oxygen and blood [95]. FUNDC1 interacts with both fission and fusion machinery components, regulating mitochondrial dynamics. Mitochondrial phosphatase phosphoglycerate mutase 5 (PGAM5) dephosphorylates FUNDC1, thereby disrupting its physical association with OPA1, and inhibiting mitochondrial fusion under hypoxic conditions. In turn, FUNDC1 translocates to ER–mitochondrial contact sites, mediating DRP1 recruitment and mitochondrial fragmentation. Thus, FUNDC1 coordinates mitochondrial morphology and mitophagy under stress. FUNDC1 may also serve as an ULK1 adaptor; their interaction promotes ULK1 relocation on mitochondria, allowing de novo phagophore biogenesis [96].

Taken together, the diverse repertoire of receptor and adaptor molecules highlights the existence of compensatory mechanisms that regulate mitochondrial numbers in response to environmental and/or intracellular signals. The complex interplay between mitophagy pathways ensures energy metabolism and tissue homeostasis. Thus, maintenance of mitochondrial function, through a fine-tuned mitochondrial quality control system, is critical for cellular and organismal survival [97].

#### Regulation of mitophagy: activation and inhibition

Impaired mitophagy is believed to be a key factor resulting in many pathological conditions. However, overactivation of mitophagy is also harmful for the cell hemostasis [98, 99]. Therefore, maintaining the balance of promotors and inhibitors is quite important for mitochondrial quality and cell hemostasis.

Studies aiming to discover pharmacological reagents capable of promoting the clearance of dysfunctional or damaged organelles are becoming more prevalent [100]. Positive activators of autophagy, such as rapamycin and metformin, regulate the activity of 5′ AMP-activated protein kinase (AMPK) and mammalian target of rapamycin (mTOR), and assist in preserving energy metabolism, possibly by balancing mitochondrial clearance and biogenesis [101, 102]. Rapamycin administration reportedly exerted positive effects on regulating mitochondrial quality by maintaining energy homeostasis and stress resistance in mammalian cells [103, 104]. Metformin supplementation triggers mitophagy by increasing the activity of Parkin, and by downregulating P53 levels [105]. In addition, some other natural compounds, such as urolithin A, resveratrol, and antibiotics, also maintain mitochondrial integrity by inducing mitophagy. Moreover, the mitophagy triggered by these compounds exerts protective and anti-aging effects by restoring energy hemostasis in both mammals and yeast [106–110]. PMI (p62–SQSTM1-mediated mitophagy inducer), one type of artificial chemical, can stabilize nuclear factor erythroid 2–related factor 2 (Nrf2) and induce p62-mediated mitophagy [111]. Ubiquitin Specific Peptidase 8 (USP8) is a cytoplasmic deubiquitinating enzyme (DUB), and functions as a promotor of mitophagy [112, 113]. USP8 has no effect on Parkin’s substrates. On the contrary, it deubiquitinates and stabilizes Parkin directly by removing K6-chains from Parkin [113].

In addition to promoting mitophagy, some negative regulators of mitophagy have been determined. As the induction of mitophagy largely depends on ubiquitination (such as Parkin-dependent pathway), a growing number of researchers have placed their emphasis on DUBs to downregulate mitophagy. From the ~ 80 active DUBs discovered in mammal cells [114], USP35, USP30, and USP15 exert direct deubiquitination effects on Parkin substrates, thus negatively regulating mitophagy [115]. Recently, Wang et al. [116] found that PTEN-L could act as an inhibitor of mitophagy by directly dephosphorylating Ub and Parkin.

Taken together, maintaining the balance of mitophagic promotion and inhibition is important for normal mitochondrial functions and cellular homeostasis. Studies attempting to discover new compounds with both biogenic and mitophagic abilities provide promise for developing novel therapeutic strategies on mitochondrial diseases [117]. Besides, the post-translational modifications for mitophagy can be concluded as ubiquitination/deubiquitination, acetylation/deacetylation, and phosphorylation/dephosphorylation [118].

### Pexophagy

#### General introduction of peroxisome and pexophagy

Peroxisomes are heterogeneous and dynamic organelles, and vary in number, size, and function among different cell types and metabolic status. This versatile organelle primarily functions to degrade fatty acids, such as very-long-chain and polyunsaturated fatty acids, and metabolize reactive oxygen species (ROS) [119–122]. Peroxisome homeostasis depends on the balance between the degradation and biogenesis of peroxisomes in different physiological contexts. Any disturbance of the integrity and number of peroxisomes would disrupt the homeostasis of cells, leading to cell death. Therefore, the selective removal of superfluous and damaged peroxisomes, known as pexophagy, is critical to maintain redox homeostasis [123].

The term pexophagy was first described by Klionsky in 1997 [124]. Later, researchers found that pexophagy could be divided into two modes, macropexophagy and micropexophagy [125–127]. In mammals, macropexophagy means that a single peroxisome is engulfed by autophagosomes to form a pexophagosome, which is fused with lysosomes and then degraded for recycling. In micropexophagy, the peroxisome is engulfed by vacuolar sequestering membranes (VSMs) and micropexophagy-specific apparatus (MIPA) [128], which forms a lid over the cup-shaped VSMs cradling the peroxisomes [129].

Except for the proteins of core autophagy machinery, many specific proteins are reportedly involved in the process of pexophagy, such as autophagy receptors (Fig. 2b). In mammalian cells, the NBR1 and SQSTM1/p62 are reportedly the autophagy receptors for pexophagy [130]. These receptors share two similar functional domains. For example, LIR binds to LC3, and thus delivers peroxisome to the autophagosome. The other is a ubiquitin-associated domain that allows itself to interact with ubiquitinated residues on the peroxisome [131]. Although SQSTM1 plays an important role in pexophagy, it is not required for pexophagy when NBR1 is sufficient. However, SQSTM1 can increase the efficiency of NBR1-mediated pexophagy by binding with NBR1 [132]. Additionally, these two receptors are not only specific for pexophagy, they are also reported to participate in mitophagy, lysophagy, and ER-phagy [133–135]. Besides, PEX14 is reportedly involved in the pexophagic process by directly interacting with LC3-II under nutrient starvation [136]. NBR1 and/or SQSTM1/p62 reportedly facilitate the interaction between PEX14 and LC3-II by inducing a conformational alteration of PEX14, which allows LC3-II to interact with the transmembrane domain of PEX14 [137]. Recent studies show that PEX5 ubiquitination is an important mechanism in initiating pexophagy in response to some stresses, such as peroxisomal dysfunction or oxidative stress. Furthermore, another important factor is ataxia-telangiectasia mutated (ATM) kinase. Activation of ATM could phosphorylate and activate PEX5, which leads to PEX5 self-ubiquitination and pexophagic promotion [138, 139].

#### Molecular mechanisms of pexophagy

##### Ubiquitination-mediated pexophagy

Growing evidence has shown that ubiquitination of some specific proteins is the requisite for selective autophagy [45, 140, 141]. Recently, PEX5 ubiquitination is found to be a key role in the pexophagy. Oxidative stress signaling phosphorylates and activates peroxisome-localized ATM, which activates PEX5 via phosphorylation at S141. Then, phosphorylated PEX5 can be ubiquitinated at K209 by the peroxisomal E3-ligases PEX2/10/12 and recognized by SQSTM1/p62, which targets peroxisomes for pexophagy [138].

##### Adaptor-mediated pexophagy

SQSTM1/p62 acts as an autophagy adaptor and has two important functional domains: LIR of the motif and a UBA domain at the C-terminus [134, 142]. As an autophagy adaptor, SQSTM1/p62 is the regulatory center for autophagic signaling pathways, and is always adapted as a biomarker for evaluating the level of autophagy [134, 143, 144]. In the process of pexophagy, the LC3-interacting region (LIR) of SQSTM1/p62 binds with LC3-II, and the Ubiquitin-Associated (UBA) domain connects with ubiquitinated regions of peroxisomes, resulting in pexophagy and engulfment of the peroxisome [145, 146].

NBR1 is another mediator for pexophagy. NBR1 also contains LIR at the center of the protein and a UBA domain at the C-terminus [147, 148]. NBR1 upregulation reportedly promotes pexophagy by recruiting peroxisomes and acting as a “see me” signal to be recognized by lysosomes [132]. Besides, p62 lacks a juxta-UBA (JUBA) domain that is required for subcellular localization, but it can promote the efficacy of NBR1-induced pexophagy by interacting with NBR1 [132].

### ER-phagy (reticulophagy)

The homeostasis maintained by ER is vital for both cellular activity and cell survival. Various exogenous or intracellular stresses, such as imbalance of calcium flux, oxidative stress, protein-folding dysfunction, and disruption in ER functions, which leads to the accumulation of unfolded or misfolded proteins, causing ‘ER stress’ [149–151]. One salvage measure that responds to the ER stress is the selective degradation of misfolding proteins or the ER membrane itself. The term “ER-phagy”, also known as “reticulophagy”, was first described by Bernales et al. in 2007, who also found that ER-phagy was induced by ER stress [152].

Many researchers, inspired by the study of mitophagy and pexophagy, have sought to discover the autophagy adaptors or ER-phagy receptors (Fig. 2b). Recently, family with sequence similarity 134, member B (FAM134B) was reported to show advantages in facilitating the degradation of ER membranes [153]. FAM134B, an intramembranal ER-resident protein, is characterized by the presence of a reticulon homology domain (RHD). Khaminets et al. found that the FAM134 reticulon protein family could act as receptors to interact with LC3 or gamma-aminobutyric acid receptor-associated protein (GABARAP), and promote the turnover of ER membrane (‘ER-phagy’). Reticulon 3 (RTN3), an RHD-containing protein, is located at ER tubules, and its major function is to facilitate the formation of ER tubules [154]. Among several splicing isoforms of RTN3, only the longest one is equipped with six LIR domains, which could bind LC3/GABARAP, promote the segmentation of ER tubules, and finally lead to ER-phagy. In fact, RTN3 initiates ER-phagy mainly under conditions of energy or oxygen deprivation. However, RTN3 and FAM134B only exert their function as an ER-phagy receptor in the region they are located [155]. Additionally, a specialized ER-phagy receptor, cell-cycle progression 1 (CCPG1), was discovered, and acts in response to the massive accumulation of misfolded or aggregated proteins in the ER. CCPG1 is a transmembrane protein, and resides in the ER. In yeast, it can prevent cells from cell-cycle arrest, hence the name [156]. As an autophagy receptor, CCPG1 has an LIR motif, which binds with LC3. Moreover, CCPG1 also has an FIR motif that interacts with autophagic proteins, RB1CC1/FIP200. Binding to RB1CC1/FIP200 increases the efficiency of ER-phagy [157]. Recently, Chen et al. have identified a new ER-phagy receptor, Atlastin GTPase 3 (ATL3), which belongs to a family of dynamin-like GTPase. ATL3 has been shown to facilitate ER fusion. Besides, as an ER-phagy receptor, especially for tubular ER, ATL3 binds with GABARAP subfamily proteins through 2 GABARAP-interacting motifs (GIMs) [158].

Unlike other forms of selective autophagy, ER-phagy has its own characteristics. First, most of the ER-phagy receptors are ER-resident proteins. For example, FAM134B [153], the first ER-receptor identified in mammalian cells, is an intramembrane ER-resident protein which has an RHD at the N-terminal. Moreover, FAM134B possesses an LIR domain at C-terminal, which can bind to GABARAP/LC3. ATL3, a recently identified example of an ER-phagy receptor, has two transmembrane regions. This differs from other autophagy receptors, because the LIRs of ATL3 are specific GIMs, which can specifically bind to the GABARAP subfamily. Second, only some portions or fragments of the ER are involved in the selective degradation process, but not the entire organelle. Additionally, in other types of selective autophagy, such as mitophagy or pexophagy, the cargoes are wholly encapsulated by the autophagosome [159]. Thirdly, receptors mediate the selective autophagy of different sub-regions of the ER. For example, FAM134B primarily targets sheet-like ER for degradation, while the long isoform of RTN3 (RTN3L) and ATL3 has effects on tubular ER [153, 155, 160]. Fourth, different ER-phagy receptors exert their functions in different pathophysiological contexts. For example, RTN3, FAM134B, and ATL3 are activated by nutritional deficiencies, while CCPG1 is activated by ER stress. Finally, different ER-phagy receptors mediate ER-phagy in different cell types or tissues. For instance, FAM134B is located in embryonic fibroblasts and U2OS cells, and RTN3L is expressed in kidney and heart, while ATL3 mainly exert its functions in the absence of RTN3L [161, 162].

The ER-phagy shows its physiological value in two ways: (1) some parts of the ER, which are dysfunctional or have accumulated unfolded or misfolded proteins, could be engulfed and degraded via ER-phagy; (2) ER-phagy may represent an important response to ER stress [163].

### Aggrephagy

Protein aggregation means the accumulation of unfolded or misfolded proteins, which twine together to form insoluble clumps. The aggregates are always detrimental to the cells, and cause a series of pathological problems, including AD, PD, etc. [164–166]. To be noted, aggregation reportedly acts in a protective role for the cell by isolating damaged or dysfunctional proteins in an insoluble form [167]. In cells, three systems are responsible for the quality of proteins: chaperone-assisted folding, proteasomal-dependent degradation, and aggrephagy, a form of selective autophagy that participates in the degradation of ubiquitinated aggregates [168]. These proteins are labeled with ubiquitin (Ub), which binds to their adaptors. The subsequent process of aggrephagy can be briefly explained by the damaged or unfolded proteins that form aggregates, which is labeled by ubiquitination. Then, the aggregates are recognized and engulfed by a double-membrane to form autophagosomes. Finally, the autophagosomes fuse with lysosomes for further degradation and recycling [169].

Like other forms of selective autophagy, aggrephagy depends on the functions of adaptors, such as SQSTM/p62, NBR1, histone deacetylase 6 (HDAC6), and ALFY (autophagy-linked FYVE domain-containing protein) [170–173]. Ubiquitination of misfolded proteins is a key mediator in the recognition and degradation of protein aggregates by aggrephagy. Since all of these receptors possess one or more LC3 interaction regions (LIRs) and one ubiquitin-binding domain (UBA), the proposed role of these receptors in aggrephagy is to bridge LC3/GABARAP family members with ubiquitinated substrates [174]. In both p62 and NBR1, the UBD that is located in the C-terminal region specifically recognizes Lys63-linked polyubiquitin substrates and forms a complex [175]. Simultaneously, the LC3-interacting motif in p62 and NBR1 promotes the delivery of complexes to form autophagosomes. Among these receptors, p62 is the only essential one for the regulation of substrate ubiquitination [176]. Additionally, p62 recruits a 400 KD autophagy-linked FYVE (ALFY) nuclear protein into the cytoplasm for autophagic degradation of aggregates. ALFY is crucial in facilitating interaction between p62-linked aggregates and the membrane-bound autophagosome, LC3 [177]. The ALFY C-terminal region has BEACH, FYVE, and WD40 domains, which are crucial to this peptide’s functional role in aggrephagy [178]. In particular, the binding of its WD40 domain to Atg5 is essential for ATG5–ATG12–ATG16L1 E3 ligase complex formation. Binding of its FYVE domain to PtdIns3P enhances phagophore formation, while its BEACH domain binds to the p62-aggregate complex and acts as a scaffold between LC3 in phagophores [173]. Under normal autophagy conditions, p62 and NBR1 aggrephagy receptors facilitate aggregate degradation. Tripartite Motif Containing 50 (TRIM50) is an E3 ubiquitin ligase, and it reportedly increases the aggregation of polyubiquitinated substrates in aggresomes. It enhances aggrephagy by increasing p62 expression and by influencing HDAC6-mediated misfolded protein retrograde axonal transport when proteasomal aggregate degradation is impaired [179]. Misfolded proteins generated in axons and dendrites are retrogradely transported to the lysosome-rich microtubule-organizing center (MTOC). In MTOC, they are packed into aggresomes, and are subsequently degraded in the lysosome [180]. These functions are regulated by histone deacetylase-6 (HDAC6). HDAC6 is a deacetylating enzyme that is crucial in microtubule transport machinery [181]. In aggrephagy, HDAC6 deacetylates α-tubulin, cortactin, and HSP-90 [182]. Furthermore, HDAC6 is actively involved in the sorting of polyubiquitinated misfolded proteins for the axonal retrograde transportation that uses Dynein-snapin, a motor–adaptor complex [183].

### Others

Selective clearance of ribosome is known as ribophagy, which was first noted by Kraft et al. in 2008. They found that in the setting of nitrogen starvation, the components that formed the 60S subunit of ribosome are more likely to undergo degradation in a lysosomal-dependent manner than the control cytoplasmic proteins [184, 185]. Ubp3- and Bre5-dependent degradation of ribosomes has been observed in yeast upon starvation. In addition, Kraft et al. have reported that Rsp5 was also involved in the regulation of ribophagy; however, it was not essential. Ribophagy has recently been identified in mammalian cells by Wyant et al. [186]. Indeed, their report has revealed the presence of a putative ribosome receptor-NUFIP1, which is required for ribophagy. NUFIP1 contains an LIR motif, and it can directly interact with LC3, thereby delivering ribosome to the lysosome for degradation. However, the ribosomal factor, which is recognized by NUFIP1, has not yet been identified. Therefore, more studies are necessary to focus on exploring the underlying mechanisms of ribophagy induction and its regulatory pathway.

Recently, it was reported that the LD can also be degraded in a lysosome-dependent pathway, known as lipophagy [187]. Lipophagy refers to a process in which LDs are isolated and engulfed by an autophagosome, which then fuses with lysosomes to be degraded [188–190]. Interestingly, given that the volume of LDs is much larger (almost 200 μm) than that of lysosomes (0.1–1 μm), the autophagosome membrane always forms on the LDs surface and pinches off parts of the LD membrane to form autolysosomes [191]. Although it is regulated by hypothalamic metabolic neurons [192], as well as many other proteins, the detailed mechanisms of lipophagy regulation remain unclear. Besides, what is currently known, is that lipophagy is quite important for cellular energy metabolism.

Moreover, the lysosomal system is the major organelle to receive cargo from the phagocytic, autophagic, and endocytic pathways, and plays an important role in maintaining nutrient and energy homeostasis. Therefore, the normal function of the lysosomal system is quite important for cellular homeostasis [193]. Several studies have demonstrated the detrimental damage incurred by a dysfunctional lysosomal system, rupture of lysosome membranes in neurodegenerative disorders, infectious diseases, and tumors. Limited damages can be repaired by the endosomal sorting complex required for transport (ESCRT) machinery. Otherwise, the lysosomes will be ubiquitinated and degraded by selective macroautophagy, known as lysophagy [194, 195]. Currently, growing studies showed that the stress-induced exposure of luminal glycans to the cytosol is the critical factor to induce ubiquitination [196]. The recognition and encapsulation of ubiquitinated lysosomes by the double-membrane depends on the functions of adaptors, such as SQSTM1/p62, TAX1BP1, and NDP52 [197, 198].

## Selective autophagy in neurological diseases: friends or foes?

There is growing evidence to suggest that there is a close relation between selective autophagy and neurological diseases (Table 2). Until now, aggrephagy and mitophagy have been the most intensively investigated types of selective autophagy in neurological diseases, whereas other types are reported less. The protective or injurious effects of selective autophagy in the occurrence and development of CNS diseases differ from each other. Similarly, the role of selective autophagy in different stages during the development of CNS diseases is different, as well. Consequently, overactive or insufficient selective autophagy may damage cells. Therefore, determining how to show the protective effect of selective autophagy on tissues (cells), and avoiding or reducing its damage to tissues (cells) to the greatest extent will likely be the focus of future research by scholars.

**Table 2:** The roles of selective autophagy in neurological diseases

Types of diseases	Selective autophagy	Mechanisms
AD	Mitophagy	Inhibits Aβ and tau pathology and reverses cognitive deficits in models of AD
	ER-phagy	Promote degradation of Ab42 and AbPP
	Lipophagy	Reduce lipid droplet accumulation and decrease neuronal neurodegeneration
	Others	Clearance of accumulation of Aβ or tau
PD	Mitophagy	Impaired mitochondria and mitophagy contributes to the pathogenesis of PD
	ER-phagy	ATL3 reveals potential physiological relevance of reticulophagy in neurodegenerative diseases
	Others	Degradation of α-synuclein and Louis bodies formed aggregates
Stroke	Mitophagy	Mitophagy prevents mitochondrial production of ROS and mitochondria-mediated apoptosis, while excessive mitophagy contributes to cell death
	Pexophagy	Reducing infarct size of ischemic stroke by attenuating oxidative stress and inflammation
	ER-phagy	An important way to cope with ER stress and reduce ER-mediated apoptosis
	Lipophagy	Decrease lipoapoptosis and oxidative products by reducing polyunsaturated FAs and other excess and harmful lipids, while it can also induce pro-apoptotic signals in the adjacent cells
	Ribophagy	One mechanism is to preserve more energy for cells to go through the attack of stroke
TBI and SCI	Mitophagy	Reduces destructive cycle of mitochondrial damage, fuel deficiency, mitochondrial apoptosis and attenuates TBI-induced BBB disruption
Brain tumors	Mitophagy	Mitophagy inhibition upregulating cell death markers (Bax, Cyt-c and caspase-3). However, mitophagy can also contribute to the cell killing effects of AT 101 and enhance the temozolomide cytotoxicity of glioma stem cells
	ER-phagy	Pharmacologically induction of ER-phagy led to reduced phospholipids phosphatidylcholine (PtdCho) and phosphatidylethanolamine (PtdE) biosynthesis
Others	Mitophagy	Mitophagic PINK1/Parkin increasing improves neuroprotection in HD; clearance of dysfunctional mitochondria in motor neurons of ALS patients, and help to rebuild mitochondrial axonal transport; Reduce inflammation to counteract inflammasome activation in astrocytes in HIV patients
	ER-phagy	I1061T NPC1 is can be degraded by ER-phagy in Niemann–Pick type C disease
	Others	Decrease in both aggregated and soluble monomeric Htt species in HD

*AD* Alzheimer’s disease, *PD* Parkinson’s disease, *TBI* traumatic brain injury, *SCI* spinal cord injury, *HD* Huntington’s disease

### Parkinson’s disease

Parkinson’s disease is a neurodegenerative disease characterized by the degenerative loss of dopaminergic neurons in the pars compacta of the substantia nigra (SNpc). Its main pathological feature is the formation of eosinophilic inclusion bodies (Lewy bodies) in neurons, which are mainly composed of α-nuclear synaptic proteins [199]. The Lewy bodies are more likely to form in aggregates due to genetic variation in both types of PD, which finally disrupts the cellular homeostasis, leading to pathology [200, 201]. Moreover, cell-to-cell propagation of malformed α-synuclein causes the contamination of healthy cells [202]. Finally, patients manifest with typical motor disturbance once the pathology causes the loss of more than 50% of dopaminergic neurons in the SNpc [203]. Once chaperone-mediated or proteasomal-dependent mechanisms fail to clear the aggregates, selective autophagy can be an alternative way to clear them [204]. Pharmacological promotion of autophagy reportedly shows neuroprotective effects for PD via selective clearance of α-synuclein aggregates [205–207]. For the regulation of aggrephagy in PD, mutations in DJ-1 or alpha-synuclein (SNCA) have been indicated to suppress aggrephagy, whereas the upregulation of NBR1 and p62 promotes aggrephagy [208]. Another recent study has shown that estrogen-related receptor α (ERRα) also participates in aggrephagy by restraining autophagy flux [209]. Besides, several studies show that mitochondrial dysfunction plays a key role in the pathogenesis of sporadic PD. In the sample of PDs, researchers found that dysfunction of electron transport complexes exists in almost 25% of patients with sporadic PD [210]. As a result, some agents, such as opiate analogue 1-methyl-4-phenyl-1,2,3,6-tetrahydropyridine (MPTP) and pesticides, can disrupt the function of the electron transport chain (ETC) and trigger a Parkinsonian phenotype in the models [211, 212]. Previous reports indicated that mitochondria within the SNpc neurons are more likely to be influenced by various stresses and damages. Therefore, any mitochondrial damage and accumulation of the damaged mitochondria cause progressive damage to the mitochondria along the whole life, unless the damaged mitochondria are labeled and selectively degraded via mitophagy. The genes encoding for α-synuclein, PINK1, DJ-1, and Parkin are all involved in mitophagy, which selectively degrade the heavily damaged mitochondria, thereby avoiding their toxic accumulation [213, 214]. Consequently, any disturbance in the genetic expressions leads to mitophagic dysfunction, further causing neurodegeneration [215]. Three mechanisms are reportedly responsible for initiating mitophagy: ubiquitin-mediated, cardiolipin-mediated, and transmembrane receptor-mediated [47]. Oh et al. have reported that S-nitrosylated PINK1 (SNO-PINK1) can impair mitophagy, whereas mitochondrial insults stimulated by age- or environmental-related stresses lead to the increase of SNO-PINK1, which inhibits the activity of SNO-PINK1. Hence, the formation of SNO-PINK1 and functional disturbance of mitophagy greatly lead to the pathogenesis of PD [215]. Besides, DNA damage is very common in the progression of PD. Accumulated DNA damage leads to a series of biochemical cascades, ultimately resulting in cellular outcomes, such as mitophagy or cell death [216]. One possible way is through the ATM–AMPK axis. ATM, a master regulator of the DNA damage response, can either directly phosphorylate or activate AMPK, which can further promote mitophagy through phosphorylation [217]. Similarly, Chen et al. [158] revealed that ATL3 is the receptor and promotor of ER-phagy, as well as the mediator of ER fusion through its specific binding to GABARAP subfamily proteins, which suggests the potential role of ER-phagy in neurodegenerative diseases.

### Alzheimer’s disease

Alzheimer’s disease (AD), a type of neurodegenerative diseases, is the major reason of dementia worldwide. The main clinical manifestations of AD typically begin with memory loss, but later presents with defects in cognitive and adaptive functioning [218–220]. AD is pathologically characterized by neuronal loss, intracellular deposits of hyper-phosphorylated tau protein, and the accumulation of amyloid-β (Aβ) in cerebral vasculature and brain parenchyma [221]. In addition, emerging evidence indicates that dysfunctional ER and mitochondria play a key role in the pathogenesis of AD [222–224].

The underlying molecular mechanisms of AD are still far from understood, but aggrephagy disturbance was reported to be a critical mechanism of AD. For example, the autophagy–lysosome pathway has been underlined as an important point for Aβ clearance [225]. In normal human CNS, there is no Aβ protein accumulation due to the higher rate of clearance compared to production, which reveals the critical role of autophagy for the degradation and production of Aβ protein [226–228]. Moreover, growing evidence suggests that there is dysfunction of the autophagy–lysosomal system in early stages of AD (without accumulation of neurofibrillary tangles or Aβ proteins), and thus, normal maintenance of autophagy has been considered a promising regimen for treating AD [228].

It is well established that mitophagy is critical for degrading dysfunctional mitochondria and maintaining mitochondrial homeostasis, a critical process for normal neuronal function. Conversely, defects in mitophagy lead to AD [229–231]. In line with these, several studies have reported that dysfunctional and damaged mitochondria were found in the brain tissues from both AD patients and animal models [229, 232]. A deficiency in mitophagic activity has been shown in a Presenilin1 mutant AD model, which directly shows the potential role of mitophagy in AD [233]. Experimental induced mitochondria dysfunction using treatment with agents or genetic alteration aggravates the manifestation of AD by increasing the deposit of Aβ and pTau aggregation [234–237]. The inhibitory role of mitophagy in the accumulation of Aβ and tau proteins has been reported by Fang et al., resulting in reversed cognitive deficits in models of AD. Resultantly, impaired removal of defective mitochondria has been suggested to be a critical factor in the pathogenesis of AD, indicating that mitophagy may be a promising therapeutic strategy [230]. Besides, it was reported that mitophagy [238] and DNA damage [239] have been closely associated with the development and progression of AD [240]. AD also shows a reduction in base excision repair (BER) [241] and double-strand breaks (DSB) [242] repair, which can be sensed by DNA-damaged sensors, such as ATM or cGAS-STING pathway, which further activates mitophagy by modulating its downstream targets [243].

Similar to PD, aggrephagy has an important role in clearing the abnormal proteins found in AD. It was reported that decreased Beclin1, defects in the lysosomal system, massive deposits of Aβ or tau proteins, and phosphorylation of P62 all decrease the effects of aggrephagy and contribute to AD [244–249].

As mentioned above, any stress causing the massive deposit of abnormal proteins in the ER lumen causes ER stress. The cells increase its protein-folding ability to deal with mild ER stress, but once this fails, the cells turn to autophagy for help [250, 251]. The earliest evidence that ER participates in autophagy was from a report, suggesting that ER acts as the source of autophagosome membrane [152]. However, ER can also be degraded by autophagy. The induction of severe ER-stress activates selective autophagy of ER, known as ER-phagy [152, 252]. The main function of ER-phagy is to isolate and degrade some parts of the ER with abnormal aggregates, which cannot be handled by other methods. What is the relationship between ER-phagy and AD? Accumulating evidence has shown that UPR activation markers are extensively increased in the brain tissues from AD patients and animal models [253, 254]. Lai et al. [255] also indicated that deposition of Aβ proteins inhibits the interaction between ER and microtubules in the hippocampal neurons, which causes the dysfunction of ER and activation of the lysosomal–autophagy system. Besides, chemicals that interfere with cholesterol metabolism within the ER reportedly increase the efficiency of Aβ42 clearance by autophagy, further indicating the close relationship between ER dysfunction and autophagy in AD [256]. Moreover, initiation of ER stress by with agents triggers autophagy, and greatly decreases the amount of mature APP and amyloid beta precursor-like protein 1 (APLP1), while autophagic inhibition contributes to the deposition of amyloid precursor protein (APP) protein and APLP1 [257]. In addition, tau protein also assists in understanding the relationship of ER and autophagy in AD. Loewen and Feany [258] have found that induction of ER stress can sometimes reduce the harmful effects of tau via introduction of autophagy.

LDs are abundant in the neurons. They mainly include glycerophospholipids, sphingolipids, and cholesterol. The normal metabolism of these lipids is quite important for the maintenance of neuronal functions. For example, cholesterol forms the main components of cell membranes and myelin, which is critical for synapse, dendrite formation [259, 260], and axonal guidance [261]. Several studies show that decreased cholesterol in neurons greatly affects neuronal activity and neurotransmission, causing the progressive degeneration of dendritic spine and synapse, which contributes greatly to the pathogenesis of AD [262–264]. Besides, sphingomyelinases have been shown to increase neuronal apoptosis by generating the pro-apoptotic molecule, ceramide [265, 266]. Besides, the level of arachidonic acid increases in the brain tissue from AD model [266, 267]. Therefore, selective autophagy of dysfunctional lipids is another key therapeutic target in treating AD. Importantly, although enhancing lipophagy has previously been indicated to reduce the accumulation of lipid droplets, and thus decrease neuronal neurodegeneration caused by the accumulation of dihydroceramide desaturases [268], there is a lack of literary evidence supporting the direct relation between lipophagy and AD. Therefore, future studies are warranted to understand the relationship between lipophagy and AD.

### Stroke

Stroke, one of the most common types of neurological diseases, is an acute cerebrovascular incident caused by either a sudden rupture of blood vessels feeding the brain (hemorrhagic stroke) or a failure of blood flow into the brain due to an abrupt blockage of blood vessels (ischemic stroke) [269]. Mitochondrial dysfunction contributes greatly to brain injury after stroke, as the mitochondria are the energy suppliers and important organelles in the regulation of oxidative metabolism and cellular apoptosis. As mentioned above, mitophagy is responsible for controlling the number and quality of mitochondria by degrading damaged and accumulated mitochondria. Besides, mitophagy is also important for maintaining the physiological functions of mitochondria, such as mitochondrial fusion and fission, or oxidative metabolism. However, the exact molecular mechanisms regarding the involvement of mitophagy in stroke remain unclarified [270]. During ischemic stroke, mitophagy is critical for maintaining normal function of the mitochondria, while aggressive mitophagy leads to cell death. Shi et al. [98] have reported that NIX mainly controls the basal level of mitophagy in physiological conditions, while BNIP3 can induce excessive mitophagy. The contribution of 12/15-lipoxygenase (LOX) to the disease pathogenesis through the increase of oxidative stress-related injury has been implicated, particularly in stroke. Besides, 12/15-LOX knockout has been shown to lead to increased macroautophagy, mitophagy, and pexophagy. It is widely accepted that inhibition of LOX provides protective effects in many diseases caused by ischemia or oxidative stress, and has also been proposed to be the culprit behind enhanced macroautophagy in the absence of LOX [271]. Next, γ-aminobutyric acid (GABA), the primary inhibitory neurotransmitter, has been shown to inhibit selective autophagy pathways, as well as mitophagy and pexophagy in yeast through Sch9, which is the homolog of S6K1, a mammalian kinase associated with oxidative stress [272]. Pexophagy, the selective degradation of dysfunctional or superfluous peroxisomes, is another selective type of autophagy that is essential for the maintenance of a balanced cellular redox state. After the stroke attack, the peroxisome population greatly increased, including the dysfunctional ones, and both lead to serious oxidative stress, neuroinflammation, and, ultimately, neuronal death. Therefore, selective autophagy of dysfunctional or superfluous peroxisomes is a key target in alleviating brain injury after stroke. Although Zhu et al. have suggested that pexophagy can exhibit neuroprotective effects by reducing the infarct size after ischemic stroke [273], the particular role of peroxisomes and pexophagy in stroke has been grossly underestimated thus far. Therefore, more studies should be carried out to explore the important role of peroxisomes in stroke.

The association between stroke and lipids, especially triglyceride and low-density lipids (LDLs), has been previously confirmed by prospective studies [274, 275], revealing that high triglyceride and ox-LDL levels substantially increase the risk of death and poor functional outcomes, both before and after stroke. Stroke is associated with a series of pathophysiological consequences, including apoptosis, inflammation, oxidative stress, and disruption in lipid metabolism. The alterations in lipid metabolism influence the amount of fatty acids (FA) and neutral lipid storage [276]. Massive accumulation of LDs is detrimental to homeostasis, as accumulation of neutral triglycerides (TGs) always promotes the metabolism of long-chain FA, which contributes to lipoapoptosis [277, 278]. Besides, oxidative stress increases the metabolism of polyunsaturated FAs (PUFAs), whose products, such as malondialdehyde (MDA), can aggravate oxidative stress [279, 280]. Kirac et al. [281] showed that ischemia and reperfusion in the liver increase lipid-mediated inflammation. Therefore, selective degradation of these types of superfluous and harmful lipids, namely lipophagy, can substantially reduce the brain injuries after stroke. Indeed, ischemic stroke reportedly induced the activation of lipophagy that occurred to effectively remove the lipid excess, modulate the lipid homeostasis, and counteract the intracellular TG overload [282]. However, induced excessive lipophagy can be a doubled-edged sword, because some products of LDs, such as ceramides and PUFA-derived lipid mediators, are detrimental to the adjacent cells [283]. Nevertheless, more research is necessary to determine the exact functions of lipophagy in stroke.

In recent years, ER-stress has been shown to play an important role in the pathogenesis of stroke. Insults that disturb ER function result in ER-stress or ER dysfunction. Hence, selective autophagy of dysfunctional ER can be a promising target to reduce injury caused by stroke. Moreover, selective isolation and engulfment of the ER assist cells in dealing with severe ER stress, even without degradation by vacuolar proteases, suggesting that selective sequestration of the ER is a critical mechanism for the cells to go through ER stress [284]. Interestingly, the study of Carloni et al. [285] found that upregulation of ribophagy exerts neuroprotective effects for animals with neonatal hypoxic-ischemia. This can be explained by the reduction of ribosome biogenesis and protein translation, which can preserve more energy for cells to endure the damage induced by stroke.

### Traumatic brain injury

Traumatic brain injury (TBI) is typically caused by an external mechanical force. The brain injuries caused by TBI can be divided into two categories: primary brain injury that occurs at the time of the insult, and secondary brain injury, which includes neuronal apoptosis, inflammation, oxidative stress, etc. [286]. Selective autophagy has been implicated in traumatic brain injury (TBI), as well.

Numerous studies have revealed that mitochondria might be critical for the pathophysiology of TBI. As the main site of energy production and oxidative metabolism, any disturbance of mitochondrial functions would lead to fuel deficiency, oxidative stress, and even induction of apoptosis, which is the key process of neural damages in TBI [287, 288]. Hence, mitophagy can alleviate secondary brain injuries by selective degradation of damaged mitochondria after TBI [289]. Wu et al. [290] have used mitochondrial division inhibitor-1 (Mdivi-1) to inhibit the key regulator of mitochondrial fission, Drp1, and have reported that it can extenuate TBI-induced blood–brain barrier (BBB) disruption and cell death by inhibiting dysfunctional autophagy, but by activating mitophagy. Likewise, Liu et al. [291] have reported increased mitophagy after TBI, which diminished the TBI-mediated intestinal epithelial cell damage, and improved intestinal permeability via ERK/Nrf2/HO-1 signaling. Mitophagy can negatively regulate IL-1β secretion, and thus inflammatory activation, to protect against TBI-triggered immunopathology [289]. The neuroprotective effects of mitophagy have also been demonstrated in spinal cord injury, which can be induced by inhibition of miRNA-124 or autophagy inducers, such as rapamycin [292–294].

### Others

In addition to the aforementioned neurological diseases, selective autophagy has been implicated to play an important role in other neurological diseases as well, such as Huntington’s disease (HD), amyotrophic lateral sclerosis (ALS), and infectious diseases of the CNS.

Huntington’s disease is an autosomal dominant neurodegenerative disease characterized by motor and cognitive impairment. The marked pathological features of HD include the formation of Huntingtin (Htt) aggregates and inclusions, which are mainly composed of Htt fragments with prolonged polyglutamine sequences (PolyQ). Some researchers have demonstrated that aggrephagy initiation greatly decreases the level of aggregated and soluble monomeric Htt species [295, 296]. Moreover, K63-ubiquitinated Htt has been shown to facilitate the target of aggregates by autophagy receptors, such as optineurin or p62 [297–299]. Besides, in preclinical HD models, growing studies have pointed to the role of mitochondrial Htt (mHtt) in mitochondrial functions and mitophagy [300]. Similarly, Valosin-containing protein (VCP), a mHtt-binding protein, is reportedly recruited to the mitochondria, leading to impaired mitophagy in models of HD [301].

Niemann–Pick disease type c is another rare, but fatal neurodegenerative disease, which is induced by genetic mutations in NPC1 (I1061T NPC1). The NPC1, a multipass transmembrane glycoprotein, is required for intracellular lipid delivery. Interestingly, Schultz et al. [302] suggested that I1061T NPC1 is selectively cleared by ER-phagy.

Amyotrophic lateral sclerosis is a neurodegenerative disease characterized by selective and progressive death of the motor neurons. Ubiquitinated inclusion bodies can be seen in the cytoplasm of these neuronal cells. Many studies have shown that mitochondrial dysfunction is a key factor in the pathogenesis of this disease. Studies have shown that mitophagy has a protective effect on ALS [303]. Specifically, dysfunction of mitophagy, due to ALS-associated mutants, is considered vital for causing mitochondrial dysfunction and accumulation, a prevalent feature in the motor neurons of ALS patients. Besides, aberrant mitochondrial axonal delivery is thought to be another factor contributing to the pathogenesis of ALS. Mitochondria from the soma are anterogradely transported to sites where the metabolism is vigorous, whereas abnormal functions of transportation lead to neuronal defects [304, 305]. Moreover, it is reported that autophagy was closely associated with DNA repair to promote neurodegeneration in ALS [306]. ALS can cause p62 mutations in or around the LC3 domain of p62, leading to autophagic defects and the accumulation of mutant p62, while the accumulation of p62 impairs the DNA damage response [307, 308]. Therefore, autophagy could be a promising target in treating ALS.

Mitochondrial dysfunction is also associated with infective processes [309], as the mitochondria have a number of roles in resisting bacterial infection, including the production of bactericidal ROS [310] and inflammasome activation [311]. Indeed, infections result in mitochondrial damage through an unknown mechanism, leading to the release of mitochondrial DNA (mtDNA) and mitochondrial ROS (mtROS) from the damaged mitochondria, which are thought to work as danger signals [312, 313]. As mentioned earlier, mitophagy is a regulatory mechanism of cells, which functions to eliminate damaged mitochondria to maintain mitochondrial homeostasis against stress and apoptosis [314, 315]. Among many others, HIV infection has been the most studied CNS infectious disease associated with mitophagy thus far. It is well known that HIV enters the CNS during the early stages of the infection, resulting in neurodegeneration and neurocognitive impairment. In HIV-productively infected astrocytes, mitophagy is crucial for cell death resistance. Moreover, mitophagy can reduce inflammation to counteract inflammasome activation; however, impaired mitophagy may favor inflammasome-mediated cell death in abortively infected cells [316]. In human primary neurons, HIV proteins, including gp120 and Tat, can cause neuronal degeneration, and thus, neurocognitive impairment by favoring the balance of mitochondrial dynamics toward enhanced fragmentation by activating mitochondrial translocation of DRP1 to the damaged mitochondria. Hence, a failure in completing the mitophagy process leads to neuronal damage [317].

## From bench to bedside: neuroprotection of selective autophagy

Although neuronal autophagy is greatly decreased when compared with other tissues, the normal development and functions of the CNS are more dependent on basal autophagy than that of other tissues [318]. The cellular division in the CNS is mainly located at developmental stages, and mature neurons have a limited or null potential of proliferation, which indicates that damaged organelles and misfolded proteins cannot be redistributed and removed by division, and finally accumulate in the neurons, unless they are successfully removed by autophagy [319, 320]. However, the cytoprotective role of autophagy in neuronal tissues was firmly proved by establishing CNS-specific autophagy-deficient animal models [321]. The neural tissue-specific knockout models for essential autophagy genes show significant signs of neurodegeneration, including growth retardation, progressive motor deficits, abnormal reflexes, and often premature death [322–324].

### Homeostatic and housekeeping functions of autophagy in CNS

Different from other cell types, the normal functions of neuronal cells greatly depend on basal autophagy, as they are post-mitotic and suffer from aggregation of toxic proteins and damaged organelles over an extended period [325, 326]. Basal autophagy showed an important role in the regulation of axonal, dendritic, and synaptic homeostasis [327]. For example, Komatsu et al. [324] reported that the loss of basal autophagy by specific knockout of the autophagic gene, Atg7, in Purkinje cells resulted in progressive dystrophy and degeneration of the axon terminals of these cells. In addition, Lee et al. [328] demonstrated the role of mTOR in regulating post-synaptic potentiation or depression, which suggested that the effects of autophagy are involved in synaptic plasticity. Taken together, basal autophagy is relatively active in healthy neurons and maintains homeostasis via degradation of accumulated proteins and dysfunctional cell organelles.

### Role of induced autophagy in neuroprotection

Growing evidence suggests that pharmacological induction of autophagic flux provides a promising clinical strategy for the treatment of neurological diseases. For example, rapamycin and its analogues, known as the ‘rapalogues’, reportedly enhance autophagosome formation by suppressing the functions of mTORC1, protecting against the toxicity of accumulated proteins in vitro, and substantially reducing neurodegeneration in fly and mouse models of HD and SCA3 [295, 329–331]. However, the beneficial effects of rapamycin treatment are greatly decreased in Drosophila models of HD and SCA3 when autophagy is inhibited [330, 332, 333]. Besides, virally delivered Beclin 1 reduced the neuropathology in mouse models of AD and Parkinson/Lewy body diseases [244, 334], which suggests that induction of autophagy enhances the neuroprotective effects of rapamycin. Consistently, other chemical agents capable of inducing autophagy in an mTOR-independent manner, such as carbamazepine, the molecular chaperone trehalose, the inositol monophosphatase inhibitors lithium, or valproate, increase the degradation of mutant huntingtin and protect against its toxicity in several models of neurodegeneration [335–337]. In addition, NAD+-induced mitophagy was also reported to reduce the cognitive loss of AD by enhancing the functions of sirtuins (SIRT1 to SIRT7), SARM1 (sterile alpha and TIR motif containing 1), and PARP (poly[ADP-ribose] polymerase) proteins [230]. Besides, decreased autophagy around the hematoma, exacerbation of neurological deficits, and brain edema in an intracerebral hemorrhage model with hyperglycemia indicate the beneficial role of autophagy in ICH with hyperglycemia [338]. For selective autophagy, the neuroprotective role of mitophagy has also been reported by many studies. Its underlying mechanisms include mitochondrial clearance and inhibition of downstream oxidative stress, apoptosis, and inflammation [339, 340]. One study showed that rapamycin could attenuate mitochondrial dysfunction via activation of mitophagy in experimental ischemic stroke [341]. In addition, several mitophagy-related proteins, such as beclin1 and Parkin, were all reported to be beneficial in the treatment of ischemic brain injury [342, 343]. Moreover, the neuroprotective effects of mitophagy have also been reported in hemorrhagic stroke by many studies [344, 345].

### Potential clinical values of selective autophagy in neurological diseases

Some clinical trials have been set to explore the potential therapeutic effects of autophagy in human diseases. For example, hydroxychloroquine (HCQ) is reported to be a clinically approved autophagy inhibitor, and has been used in cancer clinical trials (NCT00813423, NCT01023737, et al.) [346]. Besides, there are also some clinical trials studying the clinical significance of mitophagy and pexophagy in human diseases (NCT02472340 and NCT03856866). However, clinical translational applications of these drugs remain in the early stages. Current limitations include difficulties in methodology and selective drug development. One of the most challenging aspects regarding the translation of autophagy is the difficulty in dynamically evaluating autophagy in vivo. This limitation is quite important, as it decides the diagnosis and monitors the efficiency of any autophagy-based intervention. At the experimental level, tandem macroautophagy reporter mRFP-GFP-LC3 or intraventricular delivery of adeno-associated viruses to the brain have been reported to be useful methods in monitoring autophagy [347]. However, autophagy reporters are not available for use in the clinical setting yet. Therefore, developing methods for monitoring autophagy will also be important for the clinical translation of autophagy-based drugs.

## Conclusions and perspectives

In this review, we comprehensively discussed the underlying mechanisms of selective autophagy and its roles in neurological diseases. Basically, selective autophagy may be responsible for the organelle turnover, and it turns out to be an energy-efficient, fast, and precise way to deal with unwanted materials. Physiological selective autophagy is triggered by various stresses to maintain cellular homeostasis. Until now, a number of studies have mainly focused on the mechanisms of selective autophagy; however, some types of autophagy (ribophagy, ER-phagy, pexophagy, etc.) are still far from being understood. For example, no specific adaptors or receptors for ribophagy have been identified. Likewise, the process by which LDs are recognized and transported to the lysosomes remains unknown.

In addition to the mechanisms behind selective autophagy, we discussed the crosstalk between selective autophagy and other cellular processes, as selective autophagy exhibits a close relationship with apoptosis, neuroinflammation, oxidative stress, etc. Besides, most of the current studies focus on the regulation of non-selective autophagy, whereas activation of general autophagy is far from understanding the whole lysosomal–autophagy system in the cells [348]. Therefore, achieving control of selective autophagy may be a promising strategy in preventing and treating neurological diseases. However, understanding the mechanisms of selective autophagy that are behind neurological diseases has been limited to preclinical animal studies with no clinical evidence reported thus far. Indeed, it is mandatory to determine how selective autophagy mechanisms occur in the human body. We need to understand the underlying mechanisms of selective autophagy, and the selective autophagy-connected crosstalk mechanisms. Broader selective reagents and therapeutic targets for the manipulation of selective autophagy are necessary. Finally, further elucidation of selective autophagy, as well as its crosstalk mechanisms under pathologic neurological conditions is warranted.

## Abbreviations

AD: Alzheimer’s disease
PD: Parkinson’s disease
PINK: PTEN-induced kinase 1
OPTN: Optineurin
NBR1: Neighbor of BRCA1 gene 1
NUFIP1: Nuclear fragile X mental retardation-interacting protein 1
NDP52: Nuclear domain 10 protein 52
PAS: Phagophore assembly site
ULK: UNC51-like kinase
PI3K III: Class III phosphatidylinositol 3-kinase
LAMP2A: Lysosome-associated membrane protein 2
hsc70: Heat shock cognate 70
Hsp70: Heat shock protein 70
HIP: Hsc 70-interacting protein
HOP: Hsp70–Hsp90 Organizing Protein
TAX1BP1: Tax1-Binding Protein 1
PEX5: Peroxin 5
BBB: Blood–brain barrier
DFCP1: Double FYVE domain-containing protein
WIPI1: WD Repeat Domain, Phosphoinositide Interacting 1
ARIH1: Ariadne RBR E3 Ubiquitin Protein Ligase 1
SIAH1: Siah E3 Ubiquitin Protein Ligase 1
MUL1: Mitochondrial E3 Ubiquitin Protein Ligase 1
OPA1: Fission through optic atrophy 1
DRP1: Dynamin-related protein
AMPK: 5′ AMP-activated protein kinase
mTOR: Mammalian target of rapamycin
CMA: Chaperone-mediated autophagy
ROS: Reactive oxidative species
Nrf2: Nuclear factor erythroid 2–related factor 2
USP8: Ubiquitin-Specific Peptidase 8
LIR: LC3-interacting regions
UBA: Ubiquitin-associated
UBD: Ubiquitin-binding domain
CCPG-1: Cell-cycle progression gene 1
NIX: NIP3-like protein X
FAM134B: Family with sequence similarity 134
member B: Bcl2L13, BCL2-like 13
PGAM5: Phosphoglycerate mutase 5
TRIM50: Tripartite Motif Containing 50
BER: Base excision repair
DSB: Double-strand breaks
Bnip3: BCL2/adenovirus E1B interacting protein 3
AMBRA1: Autophagy/Beclin 1 regulator 1
FUNDC1: FUN14 domain-containing 1
PMI: p62–SQSTM1-mediated mitophagy inducer
DUB: Deubiquitinating enzyme
RNS: Reactive nitrogen species
VSM: Vacuolar sequestering membrane
MIPA: Micropexophagy-specific apparatus
ATM: Ataxia-telangiectasia mutated
RHD: Reticulon homology domain
GABARAP: Gamma-aminobutyric acid receptor-associated protein
GIM: GABARAP-interacting motif
LD: Lipid droplet
RTN3: Reticulon 3
CCPG1: Cell-cycle progression 1
ATL3: Atlastin GTPase 3
RTN3L: Long isoform of RTN3
HDAC6: Histone deacetylase 6
ALFY: Autophagy-linked FYVE domain-containing protein
ESCRT: Endosomal sorting complex required for transport
MPTP: 1-Methyl-4-phenyl-1,2,3,6-tetrahydropyridine
PARP-1: Mitochondrial intermembrane space
ALS: Amyotrophic lateral sclerosis
ERRα: Estrogen-related receptor α
GABA: γ-Aminobutyric acid
LDL: Low-density lipid
TBI: Traumatic brain injury
HD: Huntington’s disease
VCP: Valosin-containing protein
